# Multi-Omics, an Integrated Approach to Identify Novel Blood Biomarkers of Alzheimer’s Disease

**DOI:** 10.3390/metabo12100949

**Published:** 2022-10-06

**Authors:** Maxime François, Avinash V. Karpe, Jian-Wei Liu, David J. Beale, Maryam Hor, Jane Hecker, Jeff Faunt, John Maddison, Sally Johns, James D. Doecke, Stephen Rose, Wayne R. Leifert

**Affiliations:** 1 CSIRO Health & Biosecurity, Human Health Program, Molecular Diagnostic Solutions Group, Adelaide, SA 5000, Australia; 2 CSIRO Land & Water, Metabolomics Unit, Ecosciences Precinct, Dutton Park, QLD 4001, Australia; 3 CSIRO Land & Water, Agricultural and Environmental Sciences Precinct, Acton, Canberra, ACT 2601, Australia; 4 Department of Internal Medicine, Royal Adelaide Hospital, Adelaide, SA 5000, Australia; 5 Department of General Medicine, Royal Adelaide Hospital, Adelaide, SA 5000, Australia; 6 Aged Care Rehabilitation & Palliative Care, SA Health, Modbury Hospital, Modbury, SA 5092, Australia; 7 Australian e-Health Research Centre, CSIRO, Level 7, Surgical Treatment and Rehabilitation Service—STARS, Herston, QLD 4029, Australia

**Keywords:** Alzheimer’s disease, biomarkers, proteomics, metabolomics, systems biology

## Abstract

The metabolomic and proteomic basis of mild cognitive impairment (MCI) and Alzheimer’s disease (AD) is poorly understood, and the relationships between systemic abnormalities in metabolism and AD/MCI pathogenesis is unclear. This study compared the metabolomic and proteomic signature of plasma from cognitively normal (CN) and dementia patients diagnosed with MCI or AD, to identify specific cellular pathways and new biomarkers altered with the progression of the disease. We analysed 80 plasma samples from individuals with MCI or AD, as well as age- and gender-matched CN individuals, by utilising mass spectrometry methods and data analyses that included combined pathway analysis and model predictions. Several proteins clearly identified AD from the MCI and CN groups and included plasma actins, mannan-binding lectin serine protease 1, serum amyloid A2, fibronectin and extracellular matrix protein 1 and Keratin 9. The integrated pathway analysis showed various metabolic pathways were affected in AD, such as the arginine, alanine, aspartate, glutamate and pyruvate metabolism pathways. Therefore, our multi-omics approach identified novel plasma biomarkers for the MCI and AD groups, identified changes in metabolic processes, and may form the basis of a biomarker panel for stratifying dementia participants in future clinical trials.

## 1. Introduction

Alzheimer’s disease (AD) is a neurodegenerative disease and the main form of dementia, exhibiting clinical characteristics such as the decline of cognition and gradual loss of memory combined with multiple behavioural changes [1,2]. Due to the growing ageing population worldwide, the incidence of AD is expected to accelerate over the next thirty years, especially in developing countries [3]. Therefore, the impact of this disease at the societal and economic level has become a global concern [4,5,6,7]. The earlier stages of the disease, often characterised by mild cognitive impairment (MCI), is the time to intervene to modify the risk factors that can lead to cognitive decline and dementia. Approximately 14–18% of people over 70 years living with MCI develop AD annually [8,9]. Identifying individuals who are at an increased risk of developing AD would allow timely preventative intervention [10]. Unfortunately, detecting AD in the early stages has emerged as a major challenge, so the identification of innovative and reliable biomarkers is critical for disease diagnosis. Current approaches in the diagnosis of AD are inadequate and can leave patients in an unnecessarily extended state of suffering. Methods for the investigation of AD are often invasive, expensive, and cannot adequately identify biomarkers [11,12,13]. A minimally invasive approach is needed to identify individuals with symptoms reflective of the early stages of AD. Many studies looking at peripheral blood biomarkers have shown the usefulness of this approach for the diagnosis of AD [14,15]. Blood biomarkers that could identify individuals at an increased risk of developing AD and early in the stage of the disease would be useful, as this would allow early strategies to be put in place to prevent or reduce the risk of developing clinical symptoms.

Even though several hypotheses have been proposed to explain the exact mechanisms behind the cause of AD, the progression of the disease and its initial changes remain unclear and difficult to verify. Understanding biochemical changes and new biomarkers for MCI may provide a better route to target dementias, including AD, at an early phase of disease progression. Recent developments in proteomics and metabolomics methods in disease models of AD have provided valuable insights into the molecular basis of AD [16]. Targeted metabolomics in blood and saliva have also revealed new biomarkers for AD [17,18]. Furthermore, integrated metabolomic and proteomic analysis of saliva has revealed several metabolic pathways impacted by the disease and may contribute to the clinical presentation of MCI and AD [19]. Therefore, this study aimed to investigate the proteomic and metabolomic (including lipidomic) plasma signatures of cognitively normal (CN) people and people living with MCI and AD to identify specific biomarkers and metabolic pathways altered by the progression of the disease.

## 2. Materials and Methods

### 2.1. Patient Samples

This study was approved by the local Human Research Ethics Committees, CSIRO HREC 09/11), Ramsay Healthcare Ethics Committee and South Australian Health HREC. All methods were carried out in accordance with the approved guidelines, and all participants provided written informed consent before participating in the study. Diagnosis of MCI or AD was conducted by clinicians (Hecker, Faunt, Johns, and Maddison) based on the criteria outlined by the National Institute of Neurological and Communicative Disorders and the Stroke-Alzheimer’s Disease and Related Disorders Association (NINCDS-AD&DA) [20] and by using recommendations from the National Institute on Aging-Alzheimer’s Association (NI-AAA) workgroups on diagnostic guidelines for MCI [21] using clinical criteria. The diagnosis of dementia due to AD was made in the clinical setting and was consistent with “probable AD” criteria described in the guidelines paper from the NI-AAA [22].

Data reported in this study are from a total of 80 participants from the South Australian Neurodegenerative Disease (SAND) cohort [19,23], including: (1) the cognitively normal (CN) group (*n* = 40), which consisted of healthy age- and gender-matched participants; (2) the MCI group (*n* = 20), clinically diagnosed with MCI; and (3) the AD group (*n* = 20), clinically diagnosed with AD. Patients with significant cognitive comorbidities including head trauma, alcoholism, learning disability or Parkinson’s disease, were excluded from the study. Other exclusion criteria for all groups were as follows; patients who were undergoing chemotherapy/radiotherapy treatment for cancer, and those taking micronutrient supplements (folate, vitamin B12) above recommended intakes.

### 2.2. Blood Collection, Biochemical Measurements, and Statistics

Blood was collected and processed within 3 h of collection. All the samples were stored at −80 °C until analysis. Plasma vitamin B12, folate, and vitamin D were measured by the commercial clinical laboratory service, SA Pathology (Adelaide, South Australia, Australia). C-reactive protein (CRP) was measured using commercial enzymatic kits (Beckman Coulter Inc., Brea, CA, USA) on a Beckman AU480 clinical analyser (Beckman Coulter Inc., Brea, CA, USA), with CV = 2.36%.

### 2.3. Apolipoprotein E Genotyping

*APOE* genotyping for alleles *APOE*ε2, *ε*3, *ε*4 was based on allele-specific PCR methodology adapted to real-time PCR monitored by TaqMan probe [19,24].

### 2.4. Chemicals

All chemicals used were MS grade or higher, and purchased from Sigma-Aldrich (Castle Hill, NSW, Australia) unless specified otherwise. Acrylamide and Bradford assay reagents were purchased from BioRad Laboratories (Gladesville, NSW, Australia). Liquid chromatography (LC) Proteomics FlexMix Calibration Solution and Retention Time Calibration Mixture were purchased from Thermo Scientific (Woolloongabba, QLD, Australia). Gas chromatography (GC) retention time calibration standard and isotopically labelled standards were purchased from Cambridge Isotope Laboratories (Tewksbury, MA, USA).

### 2.5. Untargeted Metabolomics

Plasma samples were prepared following a previously described method [25,26]. Briefly, two aliquots of plasma (100 µL) were processed for complimentary metabolomics and lipidomics analyses. The first aliquot was prepared for untargeted metabolomics by gas chromatography-mass spectrometry (GC-MS) using three labelled internal standards (Myristic acid-D_27_ and D-Glucose ^13^C_6_ and L-Glutamine-amide-^15^N) [26]. The second aliquot was prepared for targeted metabolomics and untargeted lipidomics using liquid chromatography-mass spectrometry (LC-MS) using two internal standards (^13^C L-Phenylalanine, and ^13^C Succinic acid) [25]. 

Untargeted polar metabolite acquisition was performed on an Agilent 6890B gas chromatograph (GC) oven coupled to a 5977B mass spectrometer (MS) detector (Agilent Technologies, Mulgrave, VIC, Australia) fitted with an MPS autosampler. Qualitative identification of the compounds was performed according to the Metabolomics Standard Initiative (MSI) chemical analysis workgroup using the Qualitative Analysis software (Version B.010.00, Agilent Technologies, Mulgrave, VIC, Australia) of MassHunter workstation with standard GC-MS reference metabolite libraries (Fiehn Metabolomics RTL Library, G166766A, Agilent Technologies) and with the use of Kovats retention indices based on a reference n-alkane standard (C8-C40 Alkanes Calibration Standard, Cat. No. 40147-U). Data were processed with Mass Profiler Professional (v14.9, Agilent Technologies, Mulgrave, VIC, Australia). 

Central carbon metabolism (CCM) metabolites were analysed using the Agilent Metabolomics dMRM Database and Method [27]. Acquired CCM data were processed using MassHunter Quantitative Analysis (for QQQ) software (Version 10.0, Agilent Technologies, Santa Clara, CA, USA).

Untargeted nonpolar lipids were analysed using an Agilent 6546 liquid chromatography time-of-flight mass spectrometer (LC-QToF) with an Agilent Jet Stream source coupled to an Agilent Infinity II UHPLC system (Agilent Technologies, Santa Clara, CA, USA), as previously published [25,28]. Auto MSMS data on polled PBQC samples were obtained at collisions of 20 eV and 35 eV. The acquired MSMS lipid data were analysed using the Agilent Lipid Annotator tool (V1.0; Santa Clara, CA, USA) which assigned isometric structures based on MSMS fragmentation patterns. Annotated lipids were then curated into a PCDL, which was used to identify lipids within the remaining analysed samples with retention time thresholds (±0.15 min), MSMS spectra, and a library threshold score of more than 80%.

All metabolite and lipid sample sequences were randomly prepared and normalized to the internal standards or reference ions in preparation for downstream data analyses. A series of method blanks, non-sample matric QAQC standards and pooled biological samples were prepared and analysed randomly within each sequence batch. The residual standard deviation (RSD) of the labelled internal standards and QAQC samples was within 10%.

### 2.6. Untargeted Proteomics

Plasma samples were prepared following a modification of a previous method [19]. Plasma protein was precipitated by 4 volumes of cold acetone then air-dried before being re-dissolved into 8 M urea. Protein concentration was estimated by using Bradford assay (Quick Start™ Bradford Protein Assay Kit 2, BioRad). An aliquot of protein (5 µg) was reduced and alkylated. Proteins were then digested with trypsin and incubated at 37 °C overnight. The digestion was stopped with 1 µL of 10% (*v*/*v*) formic acid and filtered with a 0.22 µm filter. For each sample, 167 ng of the tryptic-digested peptides were injected onto the liquid chromatography-mass spectrometer (LC-MS) for analysis. Tryptic peptides (167 ng) were desalted and concentrated with a trap column (PepMap100 C18 5 mm × 300 µm, 5 µm, Thermo Scientific, Waltham, MA, USA) and separated on a nano column (PepMap100 C18 150 mm × 75 µm, 2 µm, Thermo Scientific) using an UltimateTM 3000 RSLC nano-LC system (Thermo Scientific). The eluted peptides were ionised with a Nanospray Flex Ion Source (Thermo Scientific). The spray voltage was set to 2.3 kV and the temperature of the heated capillary was set at 300 °C. After ionisation, mass spectra (MS1) and tandem mass spectra (MS/MS) analysis was performed using an Orbitrap Fusion MS (Thermo Scientific). MS survey scans of peptide precursors were performed in the Orbitrap detector, and the scan range was 400 to 1500 *m*/*z* at a resolution of 120 K (at 200 *m*/*z*). The target value of automatic gain control (AGC) was set as 4 × 10^5^. The maximum injection time for the MS was 50 ms. MS/MS was performed on the most abundant precursors of charge states 2+ to 7+ with intensity greater than 1 × 10^5^, and they were isolated by the quadrupole with a window of 1.6 *m*/*z*. Fragmentation was achieved by high-energy collisional dissociation (HCD) with collision energy of 28%. Fragments were detected in the ion trap detector in rapid scan rate mode. The AGC target was 4 × 10^3^, maximum injection time was 300 ms and the dynamic exclusion was 15 s. The instrument was set to run in top speed mode with a three-second cycle for both the MS and MS/MS scans. 

### 2.7. Protein Data Analysis

Protein Discoverer 2.2 (Thermo Scientific) and Sequest HT search engine were used to identify peptides/proteins and quantify the relative abundance of proteins. The spectrum data was searched against the UniProt *Homo-sapiens* database (Proteome ID: UP000005640, 20,311 sequences). Peptide spectral matches were validated using the Percolar algorithm, based on q-Values and 1% false discovery rate (FDR). Relative abundance was calculated from precursor abundance intensity and normalised from the total peptide amount.

### 2.8. Chemometric Analysis, Plasma Metabolome and Proteome Integration

The metabolomics and proteomics data, after a batch-effect adjustment and log transformation, were analysed using multivariate data analysis software SIMCA (version 16, Sartorius Stedim Biotech, Umeå, Sweden) and MetaboAnalyst 4.0 [29]. The Gene Ontology Resource and Enrichr were used for Enrichment analysis. The cut-off level for significant metabolites was a signal-to-noise (*S*/*N*) ratio of 10, while for proteins, it was a relative abundance of 1 × 10^5^. For statistical analysis of both metabolome and proteome, a fold change of ≤0.5 (downregulation) or ≥2.0 (upregulation), and a Benjamini–Hochberg adjusted *p*-value of ≤0.05 was used. Metabolomic and proteomic outputs were integrated using the ‘Joint-pathway analysis tool’ of Metaboanalyst 4.0 and Paintomics 3 [30], and the false discovery rate (FDR) was used to report adjusted *p*-values.

### 2.9. Statistics

Lipid enrichment analysis was performed using MetaboAnalyst 4.0 [29] and relevant pathways were identified by lipid pathway enrichment analysis using LIPEA [31], a free web tool based on the database source Kyoto Encyclopedia of Genes and Genomes (KEGG). LIPEA offers an automatic tool that emulates the ability of an expert to detect meaningful associations between lipid signatures and molecular mechanism. Assessment of the marginal means for each of the biomarkers (both proteomics & metabolomics) was assessed using generalised linear models (GLM) both unadjusted and adjusted for age, gender and *APOE* ε4 allele status. Receiver operating characteristic analyses (ROC) was performed on both each individual marker and on GLMs fitted with each biomarker of age, gender and *APOE* ε4 allele status. Multivariate selection to identify a short candidate list of biomarkers to separate CN from MCI and CN from AD was performed using the least absolute shrinkage and selection operator (LASSO). Optimal biomarker sets were then derived by minimizing the AIC and assessing multi-collinearity. Fitted values from the final models were then used in ROC analyses for each of the comparisons (CN vs. MCI & CN vs. AD) to derive a multivariate prediction for disease outcome. For many model choices, a high level of multi-collinearity meant that only one or two biomarkers could be kept along with age, gender and *APOE* ε4 allele status, otherwise the model became unstable, with beta and associated error estimates trending towards very large numbers. Where the addition of one biomarker with age, gender and *APOE* ε4 allele status created instability in the model, estimates of the biomarker alone were used for the ROC predictions. All analyses were performed in the R statistical environment, version 4.0. *p*-Values from individual biomarkers were not considered here, other than to rank the biomarkers for their largest differences between groups. Rather more importance was put on using the top biomarker set as derived from the LASSO, before fitting the final model to the ROC and deriving estimates to infer the researchers’ capability to separate CN from MCI/AD groups.

## 3. Results

### 3.1. Cohort

The demographic information of our participants, including the gender, age, mini-mental state examination (MMSE) and *APOE* ε4 allele status, is shown in Table 1. Participants with either MCI or AD were more likely to have an *APOE* ε4 allele compared with those who were CN. (*p* < 0.004). *APOE* ε4 allele status is the main genetic determinant of AD. Presence of *APOE* ε4 is associated with an increased risk of cognitive decline and AD and is an important measurement to build predictive models. Furthermore, MMSE scores showed a significant decrease with disease progression (One-Way ANOVA), whilst age and gender distribution were not significantly different across the groups.

### 3.2. Untargeted Omics

**Figure 1:**
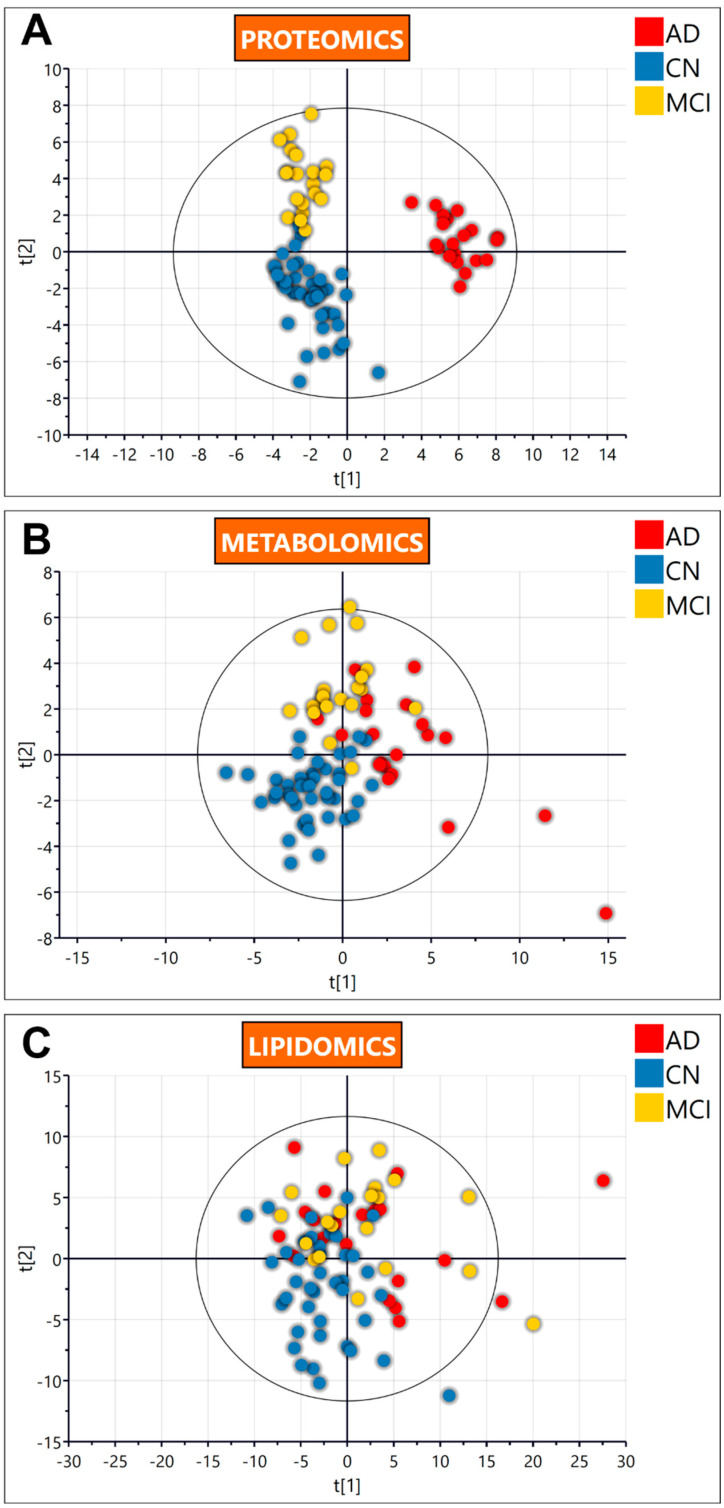
Partial least squares-data analysis (PLS-DA) score scatter plots for the proteomics (**A**), metabolomics (**B**) and the lipidomics (**C**), discriminating the three groups. A legend for the colour is provided in each plot. Abbreviations: AD, Alzheimer’s disease; CN, cognitively normal; MCI, mild cognitive impairment.

The metabolomics and proteomics analysis yielded 489 metabolites, including 251 lipids, and 168 common proteins across the three plasma sample groups collected [age-matched cognitively normal; CN (*n* = 40); mild cognitive impairment; MCI (*n* = 20), and Alzheimer’s disease, AD (*n* = 20)]. To explore variations between the groups, the metabolomic, proteomic and lipidomic data were first log-transformed, normalised and analysed by multivariate statistics. Figure 1 illustrates the partial least-squares data analysis (PLS-DA) score scatter plots for each of the data sets analysed, i.e., proteomics (Figure 1A), metabolomics (Figure 1B), and lipidomics (Figure 1C). PLS-DA scores show how the dataset can discriminate the CN, MCI and AD groups by trends based on two components. The proteomics dataset was observed to discriminate the three groups from each other. The metabolomic dataset showed some discrimination of the disease groups from the CN group. Finally, the lipidomic dataset showed some discrimination between the groups, albeit with overlap.

#### 3.2.1. Untargeted Proteomics

A total of 317 proteins have been identified, and their relative abundance levels measured in plasma. Untargeted proteomics analysis of plasma identified several proteins that showed significant differences between MCI/AD and the CN groups (at the nominal significance level only). Supplementary Table S1 lists the top 9 proteins, ranked by adjusted *p*-value, separating the MCI and the AD from the CN group. When comparing MCI and CN, Mannan-binding lectin serine protease 1 (*p* = 0.0007), haemoglobin subunit epsilon (*p* = 0.0012) and Complement C4 A (*p* = 0.0016) proteins showed the most significant differences with respect to the CN individuals. Furthermore, from the AD and CN comparison, the top 9 proteins that were most changed were extracellular matrix protein 1 (*p* = 0.0009), followed by selenoprotein P (*p* = 0.0015) and complement C4 A (*p* = 0.0017), as well as others shown in Supplementary Table S1.

In order to classify the top plasma proteins that could be considered as AD biomarkers, proteins were ranked based on their area under the curve (AUC). AUC is a value generated from an ROC curve analysis and is an indicator of the diagnostic power of a biomarker. Following this analysis, the top 9 proteins were summarised in Table 2.

**Figure 2:**
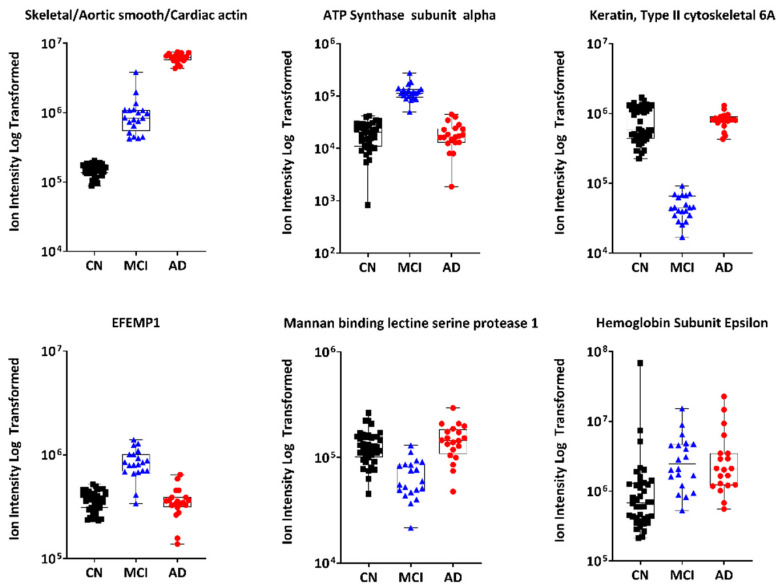
The top 6 protein biomarkers that separated the MCI group from the CN group in Table 2. The CN group is shown in black while the MCI and AD groups are shown in blue and red, respectively. Abbreviations: AD, Alzheimer’s disease; CN, cognitively normal; EFEMP1, epidermal growth factor containing fibulin like extracellular matrix protein 1; MCI, mild cognitive impairment.

**Figure 3:**
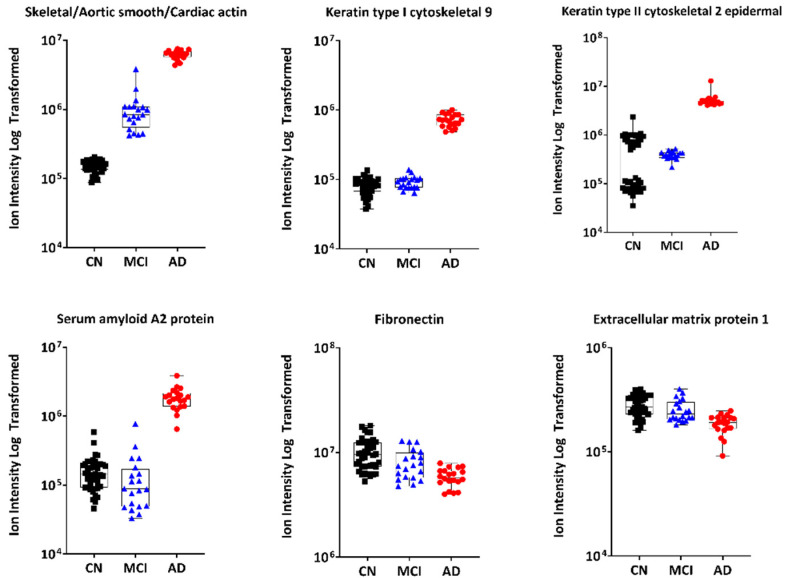
The top 6 protein biomarkers that separated the AD group from the CN group in Table 2. The CN group is shown in black while the MCI and AD groups are shown in blue and red, respectively. Abbreviations: AD, Alzheimer’s disease; CN, cognitively normal; MCI, mild cognitive impairment.

The proteomic expression indicated that some proteins are potential candidates for being MCI and/or AD biomarkers. Interestingly, skeletal/aortic smooth/cardiac actin was the strongest biomarker for both MCI and AD groups, whilst ATP synthase and serum amyloid A2 protein were specific to the MCI and AD group, respectively. To visualise the data, each of the top 6 proteins are plotted in a separate graph with the MCI group shown in Figure 2 and the AD group in Figure 3. Each of these graphs show the differences in abundance of the top proteins identified in our study between the CN, MCI and AD groups. The top-left graphs in Figure 2 and Figure 3, for instance, show the abundance level, plotted as ion intensity (log-transformed data) of skeletal/aortic smooth/cardiac actin between the three groups.

Age, sex and *APOE* genotype were incorporated into predictive proteomic models to identify which biomarkers could best identify MCI or AD patients. Predictive models were compared to the base model consisting of age, sex and *APOE* ε4 allele status variables. The base models resulted in AUC values of 0.79 and 0.72, for MCI and AD groups, respectively. The base model plus biomarker for prediction of MCI and AD diagnosis gave AUC values of 1 and *p*-values of 1.754 × 10^−10^ and 3.026 × 10^−10^, respectively.

#### 3.2.2. Untargeted Metabolomics

Similarly, as shown in Supplementary Table S2, we ranked the top 9 metabolites based on their most significantly different *p*-values in the MCI (upper panel) and AD (lower panel) groups compared to the CN individuals.

ROC curve analyses were performed on all metabolites measured in this dataset and were ranked by descending AUC (Table 3), and the top 9 metabolite markers are shown for both the MCI (upper panel) and AD (lower panel) groups. Some metabolites showed a level of separation between the groups, with AUC above 0.85. N-Acetyl-alpha-D-glucosamine-1-phosphate (AUC = 0.86) and D-Mannose (AUC = 0.85) resulted in separation between CN and MCI. For the AD group, hypoxanthine gave the highest AUC (0.86) with 90% sensitivity and 80% specificity.

Age, sex and *APOE* genotype were incorporated into predictive metabolomic models to identify which biomarkers could best identify MCI or AD patients. The base models resulted in AUC values of 0.79 and 0.72 for MCI and AD groups, respectively. The best predictive metabolomic models for prediction of MCI and AD diagnosis displayed an AUC of 0.95.

#### 3.2.3. Integrated Pathway Analysis

##### Lipidomics

**Figure 4:**
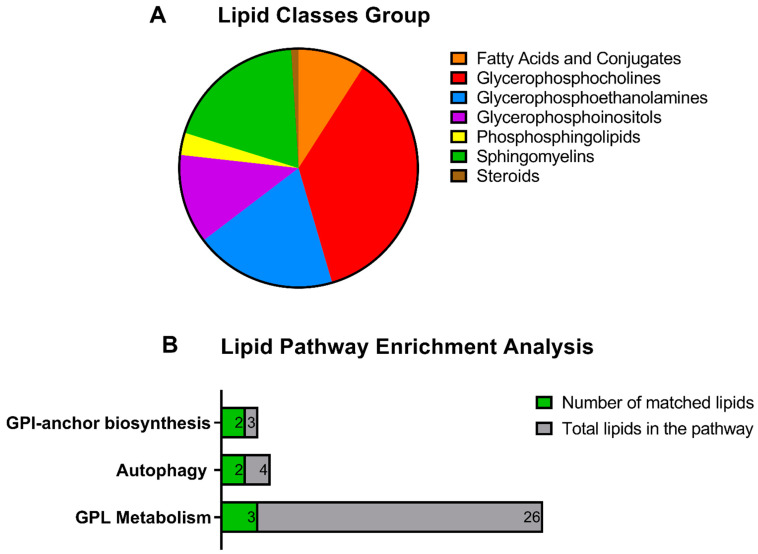
Lipid pathway enrichment analysis of plasma samples showing (**A**) the lipid classes that were detected in plasma regardless of the groups and (**B**) the lipids changing in the MCI and AD samples, representing the most impacted pathways. The green bars show the number of lipids that were measured in our sample and belonged to these pathways. Abbreviations: GPI, glycosylphosphatidylinositol; GPL, glycerophospholipid.

Among the analysed lipids, 247 were identified, belonging to 8 classes. The lipids measured were interrogated to identify biomarkers that could separate the groups (see Supplementary Table S3). Although some differences could be seen, the ROC analysis did not result in strong biomarker candidates compared to the plasma proteins and other metabolites shown earlier. To understand how the changes observed in plasma would translate at the metabolic pathway level, first a lipid enrichment analysis was carried out using Metaboanalyst to view the main lipid classes that were detected in plasma regardless of the groups (Figure 4A). The most enriched lipids measured in the samples were glycerophosphocholines, followed by sphingomyelins and glycerophosphoethanolamines. Many glycerophosphoinositols, fatty acids and conjugates were also measured, albeit less abundant. The dataset was further interrogated with a Lipid Pathway Enrichment analysis using the lipids that were identified as being altered in the MCI and AD groups. Lipid changes were linked to 19 pathways, with three of them being the most significantly associated with the changing lipids measured in our study (glycerophospholipid metabolism, autophagy and glycosylphosphatidylinositol (GPI)-anchor biosynthesis, *p* < 0.05). Two of the lipids measured in plasma samples were common to these three pathways, while three additional lipids were mapped onto glycerophospholipid metabolism (Figure 4B).

##### Pathways

**Figure 5:**
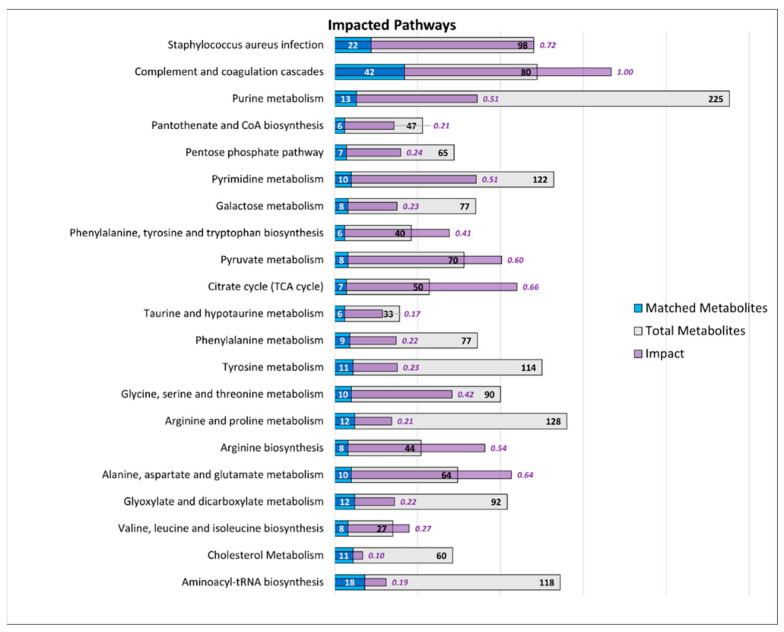
Significant pathways expressed in plasma from AD and MCI (compared with CN). The number of components of each pathway is presented as “Total metabolites” (grey bars) and the number of molecules matched to these pathways are presented as “Matched Metabolites” (blue bars). The “Impact” value represents the extent to which these pathways are impacted with the disease.

To investigate which metabolic pathways were impacted by the disease, the combined metabolome and proteome profiles of MCI and AD were integrated through the “Joint pathway analysis” tool. Results indicated the presence of 19 metabolic pathways that were significantly different (FDR *p* < 0.01) across both MCI and AD (Figure 5). Interestingly, when looking at specific pathways unrelated to metabolic processes, two pathways were found to be highly impacted by the disease (i.e., complement and coagulation cascades and *Staphylococcus aureus* infection) and are displayed at the top of Figure 5.

**Figure 6:**
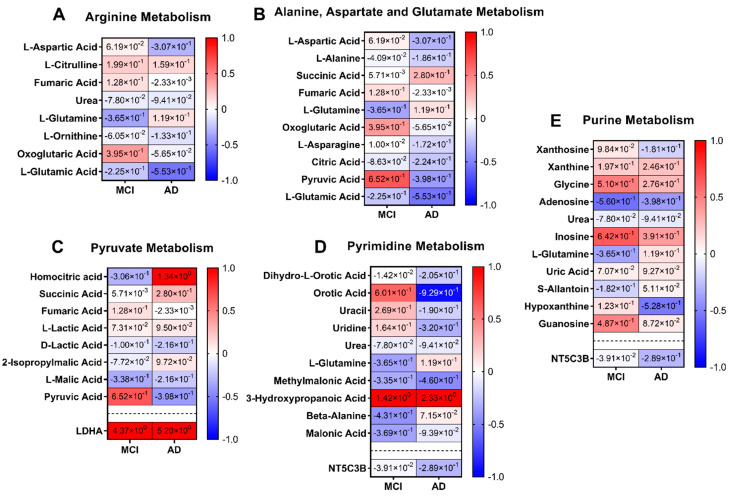
Heatmaps of metabolic pathways. Heatmaps showing metabolomic and proteomic expressions during (**A**) arginine metabolism, (**B**) alanine, aspartate, and glutamate metabolism, (**C**) pyruvate metabolism, (**D**) pyrimidine metabolism, and (**E**) purine metabolism in the plasma matrix of AD and MCI patients compared with CN individuals. The metabolites and proteins used in the multi-omics integration were selected based on the fold changes (Log_2_FC ≥ 1.00 or Log_2_FC ≤ −1.00) with statistically significant different false discovery rates (FDR ≤ 0.05)). The heatmaps were graphed by manually inputting the fold changes into GraphPad Prism 9.

Each of the mapped proteins and metabolites to the metabolic pathways have been represented as a heatmap in Figure 6; (6A) arginine metabolism, (6B) alanine, aspartate and glutamate metabolism, (6C) pyruvate metabolism, (6D) pyrimidine metabolism, and (6E) purine metabolism. Only the heatmaps of each of the mapped proteins and metabolites for the metabolic pathways that contained at least one of biomarkers listed in the top predictive models for MCI or AD are shown (Figure 6). One of the prominent observations was the accumulation of homocitric acid (Figure 6C) in the AD group (Log_2_Fold change (FC) = +1.34), while the same metabolite was decreased in the MCI group (Log_2_Fold change (FC) = −0.31). In contrast, pyruvic acid was decreased in AD (Log_2_Fold change (FC) = −0.40), while it was increased in the MCI group (Log_2_Fold change (FC) = +055). Metabolomic-proteomic integration indicated that such accumulation of the downstream products of pyruvate metabolism was likely due to upregulated lactate dehydrogenase A (LDHA; Figure 6C lower panel) activity in MCI and AD groups leading to a further imbalance in the pathway. Opposing changes in metabolites between MCI and AD were also observed in pyrimidine metabolism (Figure 6C). Oratic acid and uracil were both observed to increase in MCI (Log_2_Fold change (FC) = +0.60 and +0.27, respectively) while decreasing in AD (Log_2_Fold change (FC) = −0.93 and −0.19, respectively). Another change of note in pyrimidine metabolism was the large increase in 3-hydroxypropanoic in both the MCI and AD groups (Log_2_Fold change (FC) = +1.42 and +2.33, respectively) compared to the CN group. Furthermore, some of the top biomarkers discovered for classification of MCI and AD from Table 3 (i.e., L-glutamic acid, L-glutamine, methylmalonic acid, hypoxanthine and uridine) are linked to these impacted pathways as observed in the heatmaps (Figure 6).

## 4. Discussion

There is an urgent need for accessible and cost-effective screening tools to identify patients exhibiting early signs of neurodegeneration. Utilising current ultrasensitive mass spectrometry methods allows for the discovery of novel biomarkers of neurodegeneration in minimally invasive tissues, including plasma samples. Furthermore, advances in statistical data processing methods that combine proteomic and metabolomic pathways allow for a deeper dive into the biochemical cellular reactions impacted in MCI and AD. Our research utilised these tools to carry out plasma proteomic and metabolomic analyses to identify biomarkers for AD.

### 4.1. Lipidomics

Lipid pathway enrichment analysis was used to understand the biological pathways linked to enriched lipids measured in our study. Using LIPEA [31], we found three significantly perturbed pathways in MCI and AD, namely glycerophospholipid metabolism, autophagy, and glycosylphosphatidylinositol (GPI)-anchor biosynthesis. In the brain, a large amount of the glycerophospholipid ethanolamine are plasmalogens, a unique class of membrane glycerophospholipids and key structural phospholipids [32]. Several studies have identified a link between brain plasmalogen levels and AD. For instance, a study investigating the lipid concentration profile of post-mortem prefrontal cortex of AD brains observed differences in individual phospholipids compared to controls [33]. A significant decrease in plasmalogen choline [33] and ethanolamine [34] was observed in AD and the changes observed in the brain could reflect AD pathology around synaptic loss associated with neuroinflammation [33] or in the early stage of dementia [34]. The association between plasmalogen and AD is also reflected in peripheral tissues [35] and it is believed that age-related declines in circulating plasmalogens may increase the risk for AD [36,37]. Our results support the above, as our lipid pathway enrichment analyses highlighted glycerophospholipid metabolism as significantly impacted by MCI and AD.

Autophagy was another impacted function highlighted by our analysis which is central to the clearance of the β-amyloid and tau proteins (hallmarks proteins of AD) and displays AD-related changes before clinical hallmarks appear [38]. In fact, mouse models with impaired autophagy display exacerbated AD pathology [39]. Dysfunction of the autophagy-lysosomal system has been implicated in the pathogenesis of many neurodegenerative diseases linked to the accumulation of amyloid protein deposits, such as in AD [40].

### 4.2. Proteomics

Of 204 proteins measured, 12 were identified as biomarkers with a ROC AUChigher than 0.9. Interestingly, 6 of these proteins have been linked to the development and pathology of AD or to brain function and are discussed below.

#### 4.2.1. Plasma Actin

The most impressive marker measured in our study was the skeletal/aortic smooth/cardiac actin protein which was able to separate the three groups with 100% accuracy. Based on the highly conserved nature of these actins, we were unable to distinguish between skeletal, aortic smooth and cardiac muscle actins using our proteomic methods. Nevertheless, this protein was linearly increased across the groups and separated the CN, MCI, and AD groups from each other. A recent study demonstrated that vascular smooth muscle cells in brains of AD patients (and animal models of the disease) are deficient in contractile markers [41]. The vascular smooth muscle cell markers were correlated with Tau accumulation in brain arterioles and underwent substantial phenotypic changes in vitro under AD-like conditions, associated with a pro-inflammatory phenotype. Another study proposed that the elevated circulating concentrations of G-actin underly the post-injury reduction in DNase activity [42]. Furthermore, the chronic nature of neurodegenerative disease may initiate a compensatory Gc-globulin increase as a response to elevated levels of actin [43]. Therefore, such a biomarker that increases with the disease progression (as in the present study) is of the highest value when it comes to a diagnostic, and it calls for the pursuit of a validation study in a separate cohort.

#### 4.2.2. Serum Amyloid A2

Serum Amyloid A2 (SAA2) protein is upregulated by inflammatory cytokines and is another protein displaying a striking ability to separate the AD group, but not the MCI, with 100% accuracy from the CN group, suggesting this may not be an early biomarker of the AD process. Accumulation of SAA isoforms in the brain of AD patients has been reported [44] and intense immunostaining of SAA could be observed in affected regions of the AD brain compared to a healthy brain, particularly around myelin sheaths of axons [45]. SAA binds and transports cholesterol to aortic smooth muscle cells and thereof might have a role in cholesterol metabolism following inflammation in AD. This is of great interest as our data revealed that aortic smooth muscle protein and SAA were strongly correlated across the groups (*r* = 0.83, *p* < 0.0001), confirming a significant association of both proteins in the diseased state. Other reports suggest that SAA can promote amyloid formation in fibrinogen in blood [46], while induced expression of SAA in the brain could affect tau hyperphosphorylation [47]. In CSF, levels of SAA have been reported to be 16 times higher in AD than in a CN group [48]. It is therefore not surprising that we observed elevated peripheral levels of SAA in AD.

#### 4.2.3. Keratin 9

Keratin 9 has previously been identified as part of a protein biomarker panel of AD [49]. In that study their panel of seven proteins offered a median accuracy of 84.5% in the classification of AD cases from the CN group. A study from Richens et al. used a targeted ELISA approach and found levels of Keratin 9 in CSF and plasma were elevated in the AD group, albeit not significantly, but were significantly correlated with APOE4, clusterin and Tau levels [50]. Within our study, Keratin 9 cytoskeletal protein 1 displayed an AUC of 1 when separating the AD group from both MCI and the CN group but did not successfully separate the MCI group from the CN group. Nonetheless, the ability to segregate the AD group with 100% accuracy is impressive and highlights the diagnostic value of keratin 9 cytoskeletal protein 1 as an individual biomarker.

#### 4.2.4. Selenoprotein P

Selenoprotein P was significantly increased in both MCI (*p* < 0.004) and AD (*p* < 0.001) compared to the CN group and could separate the AD group with an AUC of 0.85. Selenoprotein P, the main transport protein of selenium, is believed to protect neuronal cells from Aβ-induced toxicity and thus prevent neurodegeneration [51,52]. Selenoprotein P gene expression was in fact found to be increased with age and, more importantly, exceeded the normal ageing baseline in AD patients [53,54]. Selenoprotein P also colocalised with Aβ plaques and neurofibrillary tangles and was reported to be increased approximately 3-fold in CSF of AD patients [55]. Our results support the case of selenoprotein P as a key protein linked to the pathogenesis of AD and, since selenoprotein is involved in selenium transport, there could be dietary implications for AD development.

#### 4.2.5. Fibronectin and Extracellular Matrix Protein 1

Relative abundance of fibronectin and extracellular matrix protein 1 (ECM1) displayed a progressive decrease from CN to MCI and AD. Both these proteins reached an AUC of 0.92 when used as single biomarkers to separate AD from the CN group. Interestingly, these two proteins are both protein components of the extracellular matrix. Although ECM1 has not been previously associated with AD, fibronectin has been investigated as a potential biomarker for AD. Expression levels of fibronectin in AD brains showed increased expression (*p* < 0.05) in the grey matter of the frontal and temporal cortex compared to healthy brains. The expression of fibronectin in the temporal cortex was further correlated with amyloid deposition [56]. In a different study, fibronectin was elevated in the plasma of MCI and AD patients compared to controls [57]. These results of a gradual decrease of fibronectin across the disease groups, while clearly indicating fibronectin as a potential biomarker of AD in blood, go in the opposite direction than those reported here. However, the specificity of isoforms measured in the blood seem to be a critical parameter in the measurements of fibronectin. High molecular forms of fibronectin, for instance, appeared more frequently in plasma of AD, and their levels were again increased compared to controls [58]. Our untargeted proteomics approach does not detail the exact isoforms identified and it is plausible that specific lower molecular forms of fibronectin are decreasing in plasma as opposed to other isoforms. A more targeted immune approach investigating all isoforms of fibronectin is required to bring clarification.

#### 4.2.6. Mannan-Binding Lectin Serine Protease 1

Mannan-binding lectin serine protease 1 (MASP-1) is one of the plasma proteins we identified as significantly decreased in the early stages of the disease, and which could be used to identify the MCI group with an AUC of 0.91. MASP-1 is an enzyme indirectly linked to AD pathology through its critical interaction with the mannan-binding lectin (MBL) protein which permits the activation of the complement system. MBL is a serum lectin and important soluble constituent of the innate immune system that forms a complex with MASP-1 in the blood. MBL has previously been measured in CSF and serum in samples from 19 AD patients and 15 controls [59]. The MBL serum levels did not differ between the groups, in contrast to the CSF levels that significantly decreased in the AD group (154 ± 35 pg/mL) compared to the control group (276 ± 50 pg/mL). Recently, MBL-binding assays conducted with or without inhibitors showed a dose-dependent increased binding of MBL to amyloid peptides (Aβ40 and Aβ42) [60]. The important role of MBL in the innate immune response, its demonstrated association with blood vessels within the brain [59] and its binding properties to amyloid, indicate a possible role in AD. However, there are still only a few investigations conducted on this specific protein as a biomarker of AD and more importantly, in the early stages of the disease in MCI.

### 4.3. Metabolomics

Untargeted metabolomics analysis of all plasma samples provided interesting insights into changes in lipids and small molecule abundance. Metabolites were weaker biomarkers compared to the protein biomarkers, based on differences between groups (*p*-Values), ROC data and statistical modelling. In fact, the top 9 metabolites for both MCI and AD all displayed ROC AUC between 0.80 and 0.87, which are not considered to be satisfactory biomarkers alone. However, when metabolites are considered collectively through the means of predictive models of the disease, biomarker panels identified MCI and AD with 95% accuracy (by ROC). This indicates that metabolite data could be used in the form of a biomarker panel in targeted approaches for AD. 

The integration of plasma metabolomic data with proteomic data allowed us to capture the overall changes in the biochemical pathways associated with disease progression. We carried out a cross-check exercise between impacted pathways and the metabolites, constituting our best predictive models. This allowed us to highlight the arginine, alanine, aspartate, glutamate, pyruvate, pyrimidine and purine metabolism pathways, as not only being impacted in MCI and AD, but also containing key metabolites of our panel of biomarkers. Our previous study investigating omics changes in this same cohort using saliva samples found that the arginine, alanine, aspartate, glutamate and pyruvate metabolisms were also significantly impacted in the MCI and AD groups [19]. Specific changes such as the accumulation of pyruvic acid in the MCI group, contrasting to a reduction in the AD group, could also be observed in plasma. A previous study examined metabolomic changes in serum of patients with AD and identified 11 metabolites as biomarkers of the disease [61]. These metabolites were found to be linked to seven biological pathways, those being arginine and proline metabolism, phenylalanine metabolism, alanine metabolism, primary bile acid synthesis, glutathione metabolism, starch and sucrose metabolism, and steroid hormone biosynthesis. Some of these were also found to be significantly impacted in our integrated plasma data overall; our metabolomics results (e.g., myo-inositol and L-glutamine) correspond well with recent studies and show that untargeted omics is a powerful tool to identify new biomarkers [62,63,64,65].

## 5. Conclusions

Proteomics and metabolomics methods can provide a relatively simple approach to identifying useful biomarkers for MCI and AD. This study provides valuable insights into the molecular basis of MCI and AD and revealed new biomarkers with several metabolomic and proteomic pathways impacted by the disease(s). Future studies should target and quantify the biomarkers so that laboratories around the world can better compare the standardised data. As such, these practical aspects should help guide a targeted approach to validating the biomarkers against other established methods including neuroimaging (for Tau and Beta amyloid), as well as other recently identified plasma biomarkers such as the various forms of p-tau, neurofilament light and GFAP, and beta amyloid (Aβ42), all of which have been used in highly sensitive Simoa assays. This study provides further support to the hypothesis that MCI and AD pathology is not only restricted to neuronal cells but also involves substantial changes in metabolic processes in the periphery.

## Figures and Tables

**Table 1:** Age and gender, MMSE scores and various blood measurements between groups.

Parameters	CN	MCI	AD	^1^*p*-Value CN vs. MCI	^1^*p*-Value CN vs. AD
Sample number	40	20	20		
Sex (F/M)	19/21	11/9	8/12	ns	ns
Age	75.3	77.8	78	ns	ns
MMSE	28.6	26.6	21.1	0.018	<0.0001
*APOE* ε4 allele positive (%) Homozygous ε4/ε4%	7/40 (17.5%) 0%	11/20 (55%) 0%	10/20 (50%) 20%		
Vitamin B12 (pmol/L)	303.8 ± 14.5	402.5 ± 42.6	395.6 ± 27.6	0.02	0.03
Folate (nmol/L)	30.1 ± 1.5	34.5 ± 2.2	33.9 ± 2.9	ns	ns
Homocysteine (µmol/L)	14.1 ± 0.6	14.1 ± 0.9	15.6 ± 1.2	ns	ns
Vitamin D3 (nmol/L)	69.1 ± 3.6	89.2 ± 5.8	70.7 ± 4.3	0.006	ns
CRP (mg/L)	2.33 ± 0.39	1.76 ± 0.39	1.41 ± 0.32	ns	ns

^1^ Bonferroni post hoc test. Abbreviations: CRP, C-reactive protein; F, Female; M, Male; MMSE, Mini-mental state examination; ns, not significant.

**Table 2:** Top protein biomarkers for clinical classification of MCI and AD adjusted for cofounders.

MCI Protein Biomarkers			
Protein	AUC (95% CI)	Sensitivity (%)	Specificity (%)
Skeletal/aortic smooth/cardiac actin	1 (1–1)	100	100
ATP synthase subunit beta	1 (1–1)	100	100
Keratin type II cytoskeletal 6A	1 (1–1)	100	100
EGF containing fibulin like extracellular matrix protein 1	0.98 (0.91–1)	95	95
Mannan-binding lectin serine protease 1	0.91 (0.84–0.99	95	82.5
Hemoglobin subunit gamma 1	0.90 (0.82–0.99)	92.5	84.2
Inter alpha trypsin inhibitor heavy chain H1	0.89 (0.81–0.97)	95	72.5
Complement C4A	0.88 (0.78–0.98)	85	82.5
Hemoglobin subunit epsilon	0.82 (0.8–0.97)	80	85
**AD Protein Biomarkers**			
**Protein**	**AUC (95% CI)**	**Sensitivity (%)**	**Specificity (%)**
Skeletal/aortic smooth/cardiac actin	1 (1–1)	100	100
Keratin type I cytoskeletal 9	1 (1–1)	100	100
Keratin type II cytoskeletal 2 epidermal	1 (1–1)	100	100
Serum amyloid A2 protein	1 (1–1)	100	100
Fibronectin	0.92 (0.86)	80	90
Extracellular matrix protein 1	0.92 (0.85–0.99)	90	85
Keratin type I cytoskeletal 16	0.91 (0.85–0.99)	100	70
Selenoprotein P	0.89 (0.82–0.97)	100	67.5
Apolipoprotein A	0.86 (0.78–0.96)	95	70

**Table 3:** Top metabolite biomarkers for clinical classification of MCI and AD adjusted for cofounders.

MCI Metabolite Biomarkers			
Metabolite	AUC (95% CI)	Sensitivity (%)	Specificity (%)
N-Acetyl-alpha-D-glucosamine-1-phosphate	0.86 (0.77–0.95)	90	70
D-Mannose	0.85 (0.76–0.94)	100	62.5
Maleic acid	0.84 (0.75–0.95)	95	70
L-Norleucine	0.84 (0.74–0.96)	85	77.5
Myo-inositol	0.84 (0.74–0.94)	90	72.5
L-Glutamine	0.84 (0.74–096)	80	80
Creatinine-1	0.84 (0.74–0.95)	95	72.5
Isopentyl acetate	0.84 (0.75–0.95)	70	90
Itaconic acid	0.84 (0.75–0.95)	70	90
**AD Metabolite Biomarkers**			
**Metabolite**	**AUC (95% CI)**	**Sensitivity (%)**	**Specificity (%)**
Hypoxanthine	0.86 (0.75–0.98)	90	80
L-Glutamic acid	0.83 (0.61–0.89)	80	75
Epinephrine	0.83 (0.73–0.93)	70	85
3-4-Dihydroxyphenylglycol	0.83 (0.73–0.94)	70	85
D-sedoheptulose-7-phosphate	0.83 (0.73–0.93)	100	57.5
N-acetyl-alpha-D-glucosamine-1-phosphate	0.82 (0.72–0.93)	85	72.5
Uridine	0.81 (0.71–0.93)	75	82.5
Methylmalonic acid	0.81 (0.71–0.93)	75	77.5
Erythrose-4-phosphate	0.81 (0.71–0.93)	87	72.5

## Data Availability

The data presented in this study are available on request from the corresponding author. The data are not publicly available due to privacy and ethics consideration.

## Supplementary Materials

The following supporting information can be downloaded at: https://www.mdpi.com/article/10.3390/metabo12100949/s1, Table S1: Top proteins different between cognitively normal, MCI and AD based on *p*-values. Abundance Data have been log transformed/normalised; Table S2: Top metabolites different between cognitively normal, MCI and AD based on *p*-values; Table S3: Top lipid biomarkers for clinical classification of MCI and AD.
