# Supplementary material for: Multi-Omics, an Integrated Approach to Identify Novel Blood Biomarkers of Alzheimer’s Disease
Source: Metabolites. 2022 Oct 6;12(10):949. doi: 10.3390/metabo12100949 (PMC9610280; doi:10.3390/metabo12100949)
Supplement: Supplementary file 1 [file metabolites-12-00949-s001.zip › metabolites-1930538-supplementary.pdf]

Supplementary Material

Table S1. Top proteins different between cognitively normal, MCI and AD based on p-values. Abundance Data have been log transformed/normalised.

**MCI protein biomarkers**				
**Protein**	**Prot ID**	**Control Mean (SD)**	**MCI Mean (SD)**	**P-value (adjusted)**
Mannan binding lectin serine protease 1	P48740.3	11.7 (0.359)	11 (0.433)	0.0007165
Hemoglobin subunit epsilon	P02100.2	13.6 (1.08)	14.7 (0.87)	0.001293
Complement C4 A	P0C0L4.2	18.2 (0.192)	18 (0.22)	0.001694
EGF containing fibulin like extracellular matrix protein 1	Q12805.2	12.8 (0.228)	13.6 (0.344)	0.002273
Hemoglobin subunit delta	P02042.2	14.1 (1.01)	15.1 (0.869)	0.00268
Selenoprotein P	P49908.3	11.7 (0.595)	12.2 (0.308)	0.004073
Inter alpha trypsin inhibitor heavy chain H1	P19827.3	16.5 (0.154)	16.7 (0.0869)	0.004114
Hemoglobin subunit beta	P68871.2	15.5 (0.834)	16.2 (0.775)	0.006025
Galectin 3 binding protein	Q08380.1	11.8 (0.358)	11.9 (0.36)	0.007708
**AD protein biomarkers**				
**Protein**	**Prot ID**	**Control Mean (SD)**	**AD Mean (SD)**	**P-value (adjusted)**
Extracellular matrix protein 1	Q16610.2	12.5 (0.242)	12.1 (0.237)	0.0009973
Selenoprotein P	P49908.3	11.7 (0.595)	12.3 (0.33)	0.001125
Complement C4 A	P0C0L4.2	18.2 (0.192)	18 (0.22)	0.001694
Tetranectin	P05452.3	13.4 (0.171)	13.2 (0.195)	0.001627
Fibronectin	P02751.4	16.1 (0.334)	15.6 (0.208)	0.002327
Pregnancy zone protein	P20742.4	12.7 (0.273)	12.9 (0.209)	0.003691
Coagulation factor X	P00742.2	9.65 (0.274)	9.34 (0.243)	0.004271
Apolipoprotein A	P02647.1	14.4 (0.565)	14 (0.517)	0.005309
Von Willebrand factor A domain containing protein 5B2	Q8N398.2	12.7 (0.458)	12.3 (0.622)	0.005337

Table S2. Top metabolites different between cognitively normal, MCI and AD based on p-values. (1), (2)Mass-spec unable to discriminate between these paired metabolites due to their similar molecular structures.

MCI metabolite biomarkers			
**Metabolite**	**Control Mean (SD)**	**MCI Mean (SD)**	**P-value (adjusted)**
N-Acetyl-alpha-D-glucosamine-1-phosphate	7.09 (0.594)	6.54 (0.841)	0.005431
L-Glutamine	9.17 (0.354)	8.86 (0.504)	0.006559
L-Norleucine	3.4 (0.257)	3.53 (0.253)	0.01747
N-Methylalanine	1.16 (0.376)	1.37 (0.28)	0.01991
3-Hydroxyisovaleric acid	1.46 (0.625)	1.02 (0.38)	0.02198
Isopentyl acetate (1)	9.84 (0.146)	9.7 (0.209)	0.0226
Itaconic acid (1)	9.84 (0.146)	9.7 (0.209)	0.0226
Myo-Inositol	15.4 (0.147)	15.5 (0.181)	0.02325
L-Fucose	0.623 (1.4)	0.13 (1.35)	0.02387
**AD metabolite biomarkers**			
**Metabolite**	**Control Mean (SD)**	**AD Mean (SD)**	**P-value (adjusted)**
L-Glutamic acid	9.98 (0.412)	9.57 (0.459)	0.003189
Hypoxanthine	9.35 (0.449)	8.73 (0.792)	0.003828
Epinephrine (2)	-2.08 (0.805)	-2.62 (0.714)	0.006018
3-4-Dihydroxyphenylglycol (2)	-2.08 (0.805)	-2.62 (0.714)	0.006018
D-Sedoheptulose-7-phosphate	5.48 (0.616)	4.95 (0.789)	0.006293
Pelargonic acid	-1.78 (0.24)	-1.67 (0.158)	0.00772
Uridine	9.61 (0.256)	9.35 (0.379)	0.009483
N-Acetyl-alpha-D-glucosamine-1-phosphate	7.09 (0.594)	6.69 (0.577)	0.01272
12a-Hydroxy-9-Demethylmunduserone-8-Carboxylic Acid	0.431 (0.241)	0.303 (0.252)	0.01306

Table S3. Top lipid biomarkers for clinical classification of MCI and AD.

MCI lipid biomarkers			
**Lipids**	**AUC**	**Fold-Change (FC)**	**P-value**
C20 H15 N2 O2	0.9825	-0.97666	1.42E-15
C26 H38 N3 O	0.96625	-0.7128	6.69E-13
C42 H85 N4 O3 Esi-23.58701	0.79125	-0.99713	0.0001
C42 H82 N2 O6 Esi-22.849985	0.75	-0.26142	0.0022
PI 18:0_22:6	0.7475	-0.68393	0.0009
C49 H80 O6	0.74625	-0.83277	0.0004
C33 H58 N3 O2	0.74	-0.42888	0.0141
C45 H79 N3 O20	0.73375	-0.36978	0.0031
PI 16:0_22:6	0.73375	-0.59816	0.0029

AD lipid biomarkers			
**Lipids**	**AUC**	**Fold-Change (FC)**	**P-value**
C5 H6 N3 O4	0.87	-0.72055	1.08E-07
C22 H48 N3 O3 S2	0.7725	0.92553	0.0004
C14 H41 N12 O8	0.77125	0.55078	0.0003
LPC 18:2/0:0	0.76	0.57328	0.0004
C19 H38 O2 Esi-11.682997	0.75125	-0.32323	0.0004
PI 16:0_22:6	0.74625	0.66571	0.0015
PC 16:0_16:0	0.73875	0.44653	0.0016
C17 H34 O2	0.73125	-0.23446	0.0101
C42 H85 N4 O3 Esi-23.58701	0.73125	0.76489	0.0042
